# Functional shoulder performance and fatigue in young adults with forward head posture with or without scapular dyskinesis

**DOI:** 10.1590/1806-9282.20260456

**Published:** 2026-09-28

**Authors:** Seda Bicici Ulusahin, Husna Balci, Nur Barlas Yalcin, Melike Nur Ozcan, Zeynep Berfu Ecemiş, Savas Kudas

**Affiliations:** 1 University of Health Sciences, Gulhane Faculty of Physiotherapy and Rehabilitation, Department of Physiotherapy and Rehabilitation – Ankara, Turkey.; 2 Cappadocia University, School of Health Sciences, Department of Physiotherapy and Rehabilitation – Nevşehir, Turkey.; 3 Cukurova University, Faculty of Health Sciences, Department of Physiotherapy and Rehabilitation – Adana, Turkey.; 4 Acıbadem Hospital, Department of Sports Medicine – Ankara, Turkey.

**Keywords:** Posture, Scapula, Shoulder joint, Fatigue, Young adult

## Abstract

**OBJECTIVE:**

The aim of this study was to compare functional shoulder performance and fatigue among young adults with forward head posture, forward head posture with scapular dyskinesis (forward head posture+scapular dyskinesia), and healthy controls.

**METHODS:**

This cross-sectional study included 85 young adults aged 18–25 years, allocated to three groups: forward head posture+scapular dyskinesia (n=25), forward head posture (n=30), and control (n=30). Forward head posture was assessed using the craniovertebral angle, scapular dyskinesia with the Lateral Scapular Slide Test, functional shoulder performance with Timed Functional Arm and Shoulder Test, and fatigue with the Chalder Fatigue Scale.

**RESULTS:**

Significant between-group differences were found for dominant-side, non-dominant-side, and total Timed Functional Arm and Shoulder Test scores (p<0.001). The forward head posture+scapular dyskinesia group had lower dominant-side and total Timed Functional Arm and Shoulder Test scores than the both forward head posture and control groups (p=0.040, p<0.001, and p=0.031, p<0.001, respectively), while both postural disorder groups had lower non-dominant-side Timed Functional Arm and Shoulder Test scores than controls (forward head posture+scapular dyskinesia: p<0.001; forward head posture: p=0.003). Total and physical fatigue scores were higher in both the forward head posture+scapular dyskinesia and forward head posture groups than in controls (total fatigue: p=0.010 and p=0.011; physical fatigue: both p=0.019), whereas mental fatigue did not differ significantly among the groups (p=0.163).

**CONCLUSION:**

Functional shoulder performance was lower in the forward head posture and forward head posture+scapular dyskinesia groups than in the controls, with the lowest scores in the forward head posture+scapular dyskinesia group. Total and physical fatigue were higher in both postural disorder groups, whereas mental fatigue did not differ between groups.

## INTRODUCTION

Forward head posture (FHP) is a common postural deviation characterized by the head being positioned forward relative to the neck in the sagittal plane^1^. FHP is characterized by excessive activity and shortening of the levator scapulae, sternocleidomastoid, upper trapezius, and suboccipital muscles. Decreased activity and muscle weakness also occur in stabilizing muscle groups such as the deep neck flexors, lower trapezius, rhomboids, and serratus anterior^2^. An imbalance between the superficial and deep muscles can lead to increased compensatory muscle activity. This can result in early fatigue and proprioceptive deficiencies^3^. FHP and scapular dyskinesia (SD) are frequently seen together, but the cause-and-effect relationship between the two situations remains unclear^4,5^. The scapula acts as a functional bridge between the neck and shoulder regions. It regulates the force transfer during glenohumeral and scapulothoracic movements, maintaining stability and mobility of the upper extremity^6–8^.

It has been reported that the coexistence of FHP and SD exacerbates the fatigue and neck and shoulder discomfort^9^. Previous studies have mainly examined FHP and SD in relation to pain, muscle performance, range of motion, and shoulder function, both individually and in combination^10,11^. However, evidence on performance-based shoulder function and perceived fatigue remains limited. Therefore, the aim of this study is to examine the effects of FHP alone and in combination with SD on functional shoulder performance and perception of general fatigue, compared with healthy individuals. We hypothesized that individuals with both FHP and SD would have lower functional shoulder performance and higher perceived fatigue compared with individuals with isolated FHP and with a healthy control group.

## METHODS

### Participants

All participants received information about the study and provided written informed consent. Inclusion criteria were age between 18 and 25 years, no history of neck, shoulder or back pain/injury, and full active shoulder range of motion. Exclusion criteria were previous orthopedic surgery involving the back, shoulder, or neck, signs of cervical radiculopathy, rheumatic disease, or pain during testing. Participants were divided into three groups: FHP, FHP+SD, and control. The study was conducted at the Gulhane Faculty of Physiotherapy and Rehabilitation Laboratory between October 2025 and January 2026, with ethical approval from the Scientific Research Ethics Committee of the University of Health Sciences (decision no: 2024-493), in accordance with the Declaration of Helsinki.

### Assessments

#### Forward head posture

FHP was assessed using the craniovertebral angle (CVA). Standardized lateral photographs were taken with participants standing relaxed and looking forward. Adhesive markers were placed on the tragus and C7 spinous process. A smartphone was mounted on a tripod at shoulder height, 1 m from the participant. Images were analyzed in Kinovea software (version 2025.1.1) by a different blinded clinician. CVA was defined as the angle between the C7–tragus line and the horizontal reference line; CVA<48° indicated FHP^12^.

#### Scapular dyskinesia

The Lateral Scapular Slide Test was performed by a blinded clinician with 18 years of experience and no role in other assessments. In three positions, the distance between the inferior angle of the scapula and the corresponding thoracic spinous process parallel to it was measured bilaterally with a tape measure. The positions were arms relaxed at the sides, hands on hips with 45° shoulder abduction, and 90° shoulder abduction with maximum internal rotation. One measurement was recorded per position, and a side-to-side difference >1.5 cm in any position was considered positive^13^.

#### Functional shoulder performance test

It was assessed using the Timed Functional Arm and Shoulder Test (TFAST), as described by Shah et al^14^. TFAST was administered by two assessors blinded to group allocation and uninvolved in clinical classification; one counted repetitions and the other timed the test. The test includes three tasks: hand-to-head and back touch, wall wash, and gallon jug lift. After a standardized demonstration, participants performed each task as quickly as possible for 30 s. In the hand-to-head and back touch task, participants stood upright with the arm at the side of the body and were asked to touch the back of the head. In the wall wash task, participants moved the hand in circular motions over a 30-cm pattern marked on the wall, in both inward and outward directions. In the gallon jug lift task, a 3.78-kg plastic jug was lifted from a counter to a shelf and returned. All tasks were performed bilaterally, with a 2-min rest between tasks, and the total score=[Hand-to-head and back+(Wall wash inward+outward)+Gallon jug lift]^14,15^.

#### Fatigue

The Chalder Fatigue Scale was used to assess fatigue. Data were collected via Google Forms in the laboratory setting on a tablet before clinical assessments. The scale consists of 11 items. The first seven items assess physical fatigue, and the remaining four items assess mental fatigue. Each item is rated on a four-point Likert scale. The total score ranges from 0 to 33. The higher scores indicate greater levels of fatigue^16^.

### Statistical analysis

Data were analyzed using International Business Machines Statistical Package for the Social Sciences Statistics 29.0. Descriptive statistics were presented as mean ± standard deviation (SD). Normality was assessed using Shapiro-Wilk, histograms, and Levene's test. Group comparisons were performed using one-way analysis of variance (ANOVA). Because six separate one-way ANOVA models were conducted for the TFAST and Chalder Fatigue Scale outcomes, Holm-Bonferroni correction was applied to the omnibus ANOVA p-values to control the family-wise type I error rate across outcome measures. For outcomes that remained significant after this correction, Tukey honestly significant difference (HSD) post-hoc tests were used. Categorical variables were compared using chi-square test.

Sample size was determined by an a priori power analysis using G*Power software (version 3.1.9.7). Based on the study by Shah et al., which established the sensitivity of the TFAST in detecting differences in functional performance, a large effect size (f=0.40) was anticipated^14^. With an alpha level of 0.05 and a desired power of 0.80, the minimum required sample size for three groups was calculated as 66. Allowing for a 10% dropout rate, a minimum of 25 participants per group was planned. The final sample consisted of 85 participants distributed across three groups (Figure 1. Study Flow). A post-power analysis was also performed with G*power. The analysis showed that the power of the study was 99% [Power (1 - β err prob)], Critical F=3.11.

**Figure 1:**
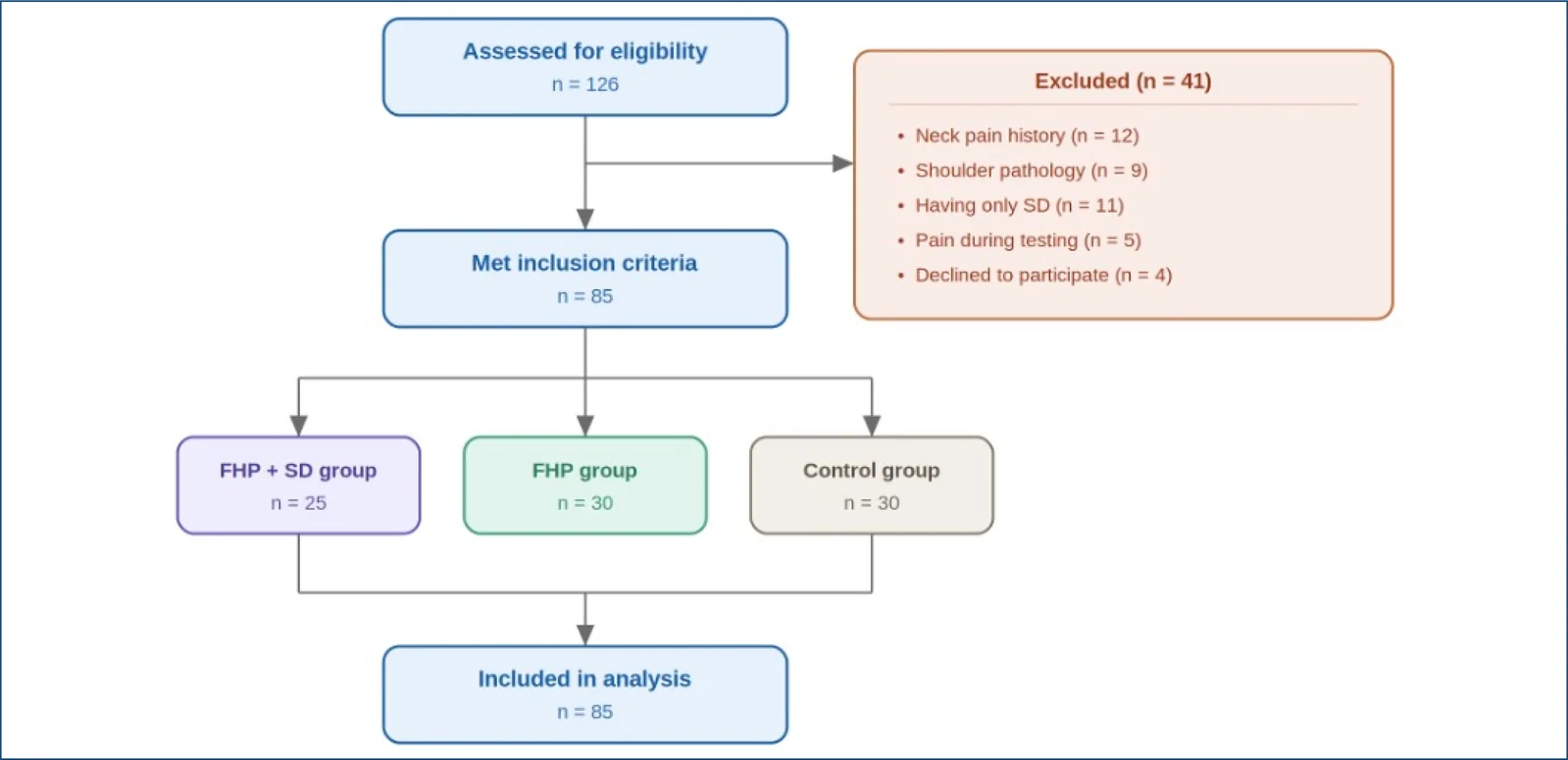
Flow diagram of participant recruitment and group classification.

## RESULTS

A total of 85 participants were included in the study: 25 in the FHP+SD group, 30 in the FHP group, and 30 in the control group. Age, sex distribution, and body mass index were similar across the groups (p=0.322, p=0.494, and p=0.220, respectively) (Table 1). After Holm-Bonferroni correction across the six omnibus ANOVA tests, significant group effects remained for dominant-side TFAST (p<0.001), non-dominant-side TFAST (p<0.001), and total TFAST scores (p<0.001), total fatigue (p=0.011), and physical fatigue (p=0.015). The group effect for mental fatigue was not significant (p=0.163).

**Table 1:** Demographic characteristics of the study groups.

Variable	FHP+SD (n=25)	FHP (n=30)	Control (n=30)	p-value
Age (years), mean±SD	21.20±1.94	21.17±1.97	20.53±1.76	0.322
Sex (female/male), n (%)	20/5 (80/20)	27/3 (90/10)	24/6 (80/20)	0.494
BMI (kg/m^2^), mean±SD	23.98±3.89	24.45±4.99	22.65±3.17	0.220

SD: standard deviation; BMI: body mass index; FHP: forward head posture; SD: scapular dyskinesia. Between-group differences assessed by one-way ANOVA (continuous variables) and chi-square test (sex).

For dominant-side TFAST, the FHP+SD group had lower scores than both the FHP groups (p=0.040) and the control group (p<0.001); the FHP group had lower scores than the control group (p=0.040). For TFAST on the non-dominant side, both the FHP+SD groups (p<0.001) and the FHP group (p=0.003) had lower scores than the control group, while the FHP+SD versus FHP comparison did not reach statistical significance (p=0.056). For total TFAST, the FHP+SD group had lower scores than the both FHP groups (p=0.031) and the control group (p<0.001); the FHP group also had lower scores than the control group (p=0.007). Both the FHP+SD and FHP groups had higher total fatigue scores than the control group (p=0.010 and p=0.011, respectively), with no significant difference between the two postural disorder groups (p=0.982). Similarly, both the FHP+SD and FHP groups had higher physical fatigue scores than the control group (both p=0.019), whereas the difference between the FHP+SD and FHP groups was not significant (p=0.990) (Table 2).

**Table 2:** Between-group comparisons of functional shoulder performance and fatigue.

Assessment parameters, mean ± SD	FHP+SD n=25	FHP n=30	Control n=30	p	Holm-adjusted p	Post-hoc Tukey HSD
TFAST score, dominant shoulder	100.40±10.84	108.83±13.92	116.87±12.46	<0.001	<0.001	1<2 p=0.040 1<3 p<0.001 2<3 p=0.040
TFAST score, non-dominant shoulder	93.56±10.63	100.67±11.58	110.60±11.29	<0.001	<0.001	1<3 p<0.001 2<3, p=0.003 1 vs. 2, p=0.056
TFAST score, total score	193.96±19.83	209.50±24.02	227.47±22.33	<0.001	<0.001	1<2 p=0.031 1<3 p<0.001 2<3 p=0.007
Chalder Fatigue Scale, total score	14.32±4.96	14.10±4.53	10.63±4.14	0.004	0.011	1>3 p=0.010 2>3 p=0.010 1 vs. 2 p=0.982
Chalder fatigue, Physical Fatigue Subscale	9.72±3.26	9.60±3.55	7.23±3.14	0.008	0.015	1>3 p=0.019 2>3 p=0.019 1 vs. 2, p=0.990
Chalder fatigue, Mental Fatigue Subscale	4.60±2.24	4.50±2.10	3.63±1.97	0.163	0.163	Not interpreted because omnibus ANOVA was not significant

SD: standard deviation; TFAST: Timed Functional Arm and Shoulder Test; FHP: forward head posture; SD: scapular dyskinesia; ANOVA: analysis of variance; Tukey HSD: Tukey's honestly significant difference test. Post-hoc: Tukey HSD. Values are presented as mean±standard deviation. Group codes: 1=FHP+SD, 2=FHP, 3=Control. Six separate one-way ANOVA models were performed; therefore, Holm-Bonferroni correction was applied to the omnibus ANOVA p-values to control the family-wise type I error rate across outcomes. Tukey HSD post-hoc tests were used for pairwise comparisons within each ANOVA and were interpreted only for outcomes with significant Holm-adjusted omnibus ANOVA results.

## DISCUSSION

In this study, individuals with forward head posture, those with both forward head posture and scapular dyskinesis, and a control group without postural deviations were compared with respect to functional shoulder performance and fatigue. According to the findings, TFAST scores were significantly lower in the FHP and FHP+SD groups than in the control group. This decrease was particularly pronounced in individuals with both FHP and SD. Furthermore, total fatigue and physical fatigue scores were significantly higher in these groups than in the control group.

To our knowledge, no study has directly compared individuals with only FHP, individuals with both FHP and SD, and healthy controls in terms of functional shoulder performance, nor has any study quantitatively demonstrated a decrease in performance. However, the literature supports that the combination of FHP and SD can negatively affect shoulder biomechanics and function by increasing muscle imbalances and postural impairment^9,11,17^. Because TFAST evaluates range of motion, endurance, and strength together, it is a suitable tool for identifying this combined effect^14^. Furthermore, it has been reported that a difference of more than 21 repetitions in this test is considered clinically significant^15^. The total TFAST difference between the FHP+SD and control groups (193.96 vs. 227.47; difference=33.51 repetitions) exceeded this threshold; however, as this value was derived from patients with shoulder problems, its relevance to asymptomatic young adults is uncertain and should be interpreted cautiously. When SD accompanies FHP, scapulothoracic mechanics may be further impaired: the scapula may not provide stable support for glenohumeral movement, the acromiohumeral distance may decrease, and trapezius-serratus anterior force balance may change^18–20^. Taken together, the lower dominant-side and total TFAST scores in the FHP+SD group suggest poorer shoulder function, although the non-dominant-side difference between FHP+SD and FHP did not reach statistical significance.

The significantly higher total and physical fatigue scores observed in both the FHP and FHP+SD groups, compared with the control group, suggest that these postural deviations may be particularly related to perceived physical fatigue. This finding is consistent with the literature reporting that forward head displacement increases the mechanical load on the cervical extensor muscles, leading to compensatory activation of the upper trapezius and levator scapulae muscles and increasing physical fatigue^2,21^. However, the lack of a significant difference between the FHP and FHP+SD groups for total and physical fatigue should be interpreted cautiously. Fatigue perception is subjective, and the absence of objective measures such as electromyography may have limited our ability to detect subtle differences between these groups. Future studies should use different fatigue scales together with objective methods to better clarify fatigue-related changes. The lack of a significant difference between the groups in mental fatigue may be due to our sample consisting of young adults aged 18–25 years. Relatively high cognitive capacity in this age group and the absence of a significant mental burden from postural compensation mechanisms may explain this situation.

This study has several limitations. First, physical activity level and screen time were not recorded; therefore, their potential confounding effects on shoulder performance and fatigue cannot be excluded. Future studies should measure and control for these variables. Second, the inclusion of only young adults may limit the generalizability of the findings to other age groups, where the cumulative effects of postural disorders on shoulder performance and fatigue may differ. Future studies should therefore include a broader age range. Although the lateral scapular slide test was applied by a single experienced clinician using a standardized clinical protocol, intra- or inter-rater reliability was not assessed in this study. Therefore, possible measurement error or misclassification of scapular dyskinesis cannot be fully excluded.

## CONCLUSION

In summary, individuals with FHP and FHP+SD exhibited significantly lower functional shoulder performance than the control group, and this impairment was more pronounced in the FHP+SD group. Total and physical fatigue scores were significantly higher in both the FHP and FHP+SD groups than in the control group, with no significant difference between the two groups. Mental fatigue scores did not differ significantly among the groups.

## Data Availability

The datasets generated and/or analyzed during the current study are available from the corresponding author upon reasonable request.
